# 5-Methylcytosine-related lncRNAs: predicting prognosis and identifying hot and cold tumor subtypes in head and neck squamous cell carcinoma

**DOI:** 10.1186/s12957-023-03067-w

**Published:** 2023-06-14

**Authors:** Juntao Huang, Ziqian Xu, Chongchang Zhou, Lixin Cheng, Hong Zeng, Yi Shen

**Affiliations:** 1 Department of Otolaryngology, Head and Neck Surgery, Ningbo Medical Center Lihuili Hospital, The Affiliated Lihuili Hospital of Ningbo University, Ningbo, Zhejiang China; 2 Department of Dermatology, Ningbo First Hospital, Zhejiang University, Zhejiang, China; 3 Department of Otolaryngology, Head and Neck Surgery, Ningbo No.2 Hospital, Ningbo, China

**Keywords:** Head and neck squamous cell carcinoma, 5-Methylcytosine methylation, Long non-coding RNA, Prognosis, Immunotherapy

## Abstract

**Background:**

5-Methylcytosine (m^5^C) methylation is recognized as an mRNA modification that participates in biological progression by regulating related lncRNAs. In this research, we explored the relationship between m^5^C-related lncRNAs (mrlncRNAs) and head and neck squamous cell carcinoma (HNSCC) to establish a predictive model.

**Methods:**

RNA sequencing and related information were obtained from the TCGA database, and patients were divided into two sets to establish and verify the risk model while identifying prognostic mrlncRNAs. Areas under the ROC curves were assessed to evaluate the predictive effectiveness, and a predictive nomogram was constructed for further prediction. Subsequently, the tumor mutation burden (TMB), stemness, functional enrichment analysis, tumor microenvironment, and immunotherapeutic and chemotherapeutic responses were also assessed based on this novel risk model. Moreover, patients were regrouped into subtypes according to the expression of model mrlncRNAs.

**Results:**

Assessed by the predictive risk model, patients were distinguished into the low-MLRS and high-MLRS groups, showing satisfactory predictive effects with AUCs of 0.673, 0.712, and 0.681 for the ROCs, respectively. Patients in the low-MLRS groups exhibited better survival status, lower mutated frequency, and lower stemness but were more sensitive to immunotherapeutic response, whereas the high-MLRS group appeared to have higher sensitivity to chemotherapy. Subsequently, patients were regrouped into two clusters: cluster 1 displayed immunosuppressive status, but cluster 2 behaved as a hot tumor with a better immunotherapeutic response.

**Conclusions:**

Referring to the above results, we established a m^5^C-related lncRNA model to evaluate the prognosis, TME, TMB, and clinical treatments for HNSCC patients. This novel assessment system is able to precisely predict the patients’ prognosis and identify hot and cold tumor subtypes clearly for HNSCC patients, providing ideas for clinical treatment.

**Supplementary Information:**

The online version contains supplementary material available at 10.1186/s12957-023-03067-w.

## Background

Head and neck squamous cell carcinoma (HNSCC), as the most recent report, appears to be an increasingly diagnosed sample of 890,000 per year and threatens human life (450,000 dead cases per year) [1, 2]. Early HNSCCs can be resected by appropriate surgery with postoperative radiotherapy, whereas when the tumor progresses to an advanced stage, the 5-year prognosis is extremely poor and the alive percentage is lower than 50% [3–5]. For patients with HNSCC, a prediction of prognosis is necessary to guide clinical treatment [5, 6]. Recently, immunotherapy has presented cheerful results in improving living conditions and prolonging the overall survival of tumor patients [7]. Among them, immune checkpoint inhibitor (ICI) therapy is used widely and commonly in the management of tumors by activating patients’ own immune defense system [8, 9]. However, few patients can gain benefits from immunotherapy due to immune escape and the complex tumor immune microenvironment (TIME) [10, 11]. For HNSCC patients, ICI therapy promotes potential therapeutic prospects and the possibility of improving prognosis; nevertheless, the individual TIME for each patient requires systematic and accurate evaluations to formulate the immunotherapeutic schedule. Therefore, it is also important and crucial to explore and develop a reliable predictive signature to assess the TIME for patients [5, 6].

5-Methylcytosine (m^5^C) methylation was considered an mRNA modification approach first reported in 1925 [12–14]. As recognized by previous studies, this RNA modification is also regulated by writers, readers, and erasers and plays an important role in biological progression by influencing RNA stability, transcription efficiency, and localization [15–17]. Reportedly, m^5^C can affect tumor progression, prognosis, and TIME as well as resistance to immunotherapy and chemotherapy [15–18]. DNMT1, as investigated by Zhang et al., could strengthen and increase the sensitivity of radiotherapeutic effects for HPV-positive HNSCC patients [19]. Additionally, compared with normal samples, NSUN2 is more enriched in tumor lesions and can significantly influence the cell cycle [20]. Similarly, long non-coding RNAs (lncRNAs) are crucial in affecting tumor progression, invasion and metastasis, and the TIME [3, 21]. Therefore, this kind of lncRNA is also considered a promising biomarker and potential target for tumor diagnosis and may provide a novel strategy to guide individualized precise treatment for tumor patients. Increasing evidence-based studies have determined that m^5^C can regulate related lncRNAs to participate and influence biological processes [15–17, 22]. Previous studies have shown that NSUN2 can alter gene and lncRNA expression as well as enhance protein synthesis and translation [14, 23]. It is recruited by the lncRNA forkhead box protein C2 (FOXC2)-AS1 and upregulated to lead a shorter survival time in HNSCC patients [15, 24]. Similarly, a significantly upregulated expression of NSUN5 was also found in tumor samples [12]; and the X-inactive specific transcript of lncRNAs can be regulated by m^5^C genes [25, 26]. It is strongly recommended that m^5^C-related lncRNAs (mrlncRNAs) be regarded as potential biomarkers to predict prognosis and immune infiltration. However, more evidence-based studies are needed to clarify the detailed mechanism and relationship among m^5^C, lncRNAs, and HNSCC.

Hence, in this study, we used bioinformatics analysis to establish a m^5^C-related lncRNA signature to predict prognosis and immune infiltration and identify tumor subtypes in HNSCC patients.

## Methods

### Obtaining the RNA-seq matrix and mrlncRNAs

Data about the RNA sequencing matrix of HNSCC was downloaded by screening The Cancer Genome Atlas (TCGA) database as fragments per kilobase million (FPKM) format, including 504 tumor tissue and 44 normally paracancerous tissue. Detailed data about clinicopathologic features and tumor-mutated frequency for each HNSCC patients were also extracted from the TCGA-HNSC cohort of the TCGA database. Subsequently, HNSCC patients from the entire cohort were equally and randomly separated into two groups (train set and test set) at a ratio of 1:1 for further model establishment and data analysis.

Additionally, according to previous studies [12–26], we obtained 15 m^5^C genes to identify their related lncRNAs, including 11 writers of *NOP2*, *NSUN2*, *NSUN3*, *NSUN4*, *NSUN5*, *NSUN6*, *NSUN7*, *DNMT1*, *TRDMT1*, *DNMT3A*, and *DNMT3B*, 2 readers of *ALYREF* and *YBX1* and 2 erasers of *TET2* and *TET3*. Furthermore, a correlation analysis of Pearson test was performed to identify relevant mrlncRNAs with the criteria of |Pearson R coefficient|> 0.04 and *p* value < 0.001 [3].

### Construction of a prognostic model and validation of predictive effects

Considering the expression of mrlncRNAs and overall survival (OS) data, univariate Cox (uni-Cox) hazard regression was performed to identify the survival-related mrlncRNAs based on the standard of a *p* value less than 0.05. Furthermore, the least absolute shrinkage and selection operator (LASSO) regression analysis was performed with tenfold cross-validation and 1000 cycles to avoid overfitting. The expression correlation between m^5^C genes and model mrlncRNAs was calculated by the Pearson correlation test and reflected in the heatmap with the application of the “limma” and “pheatmap” packages. Subsequently, the coefficient of each eligible lncRNA was calculated by multivariate Cox (multi-Cox) regression analysis, and patients in both the train and test cohorts were assessed and calculated with the following formula: m^5^C-related lncRNA risk score (MLRS) = ∑ coef (mrlncRNA)^i^ × exp (mrlncRNA)^i^, where coef means coefficient and exp means expression. Based on different MLRSs, patients were then clarified as two risk groups (low-MLRS and high-MLRS groups) concerning the median of MLRSs. The expression of model mrlncRNAs between the normal and HNSCC samples was compared, and the survival analysis was displayed referring to the best optional cutoff value. Subsequently, Kaplan‒Meier (K-M) analysis was conducted to compare the survival differences between the low-MLRS and high-MLRS groups in the test, training, and entire sets, including OS, progression-free survival (PFS), disease-free survival (DFS), and disease-specific survival (DSS). The risk score and expression of prognostic model mrlncRNAs were calculated and are shown in the plots. Furthermore, areas under the curves (AUCs) of survival receiver operating characteristic (ROC) curves about 1-, 3-, and 5-year survival status for train, test, and entire sets were calculated and compared to assess the predictive effects of the MLRS assessing system.

In addition, while performing uni- and multi-Cox survival analyses to investigate and select the independent predictive factors (*p* value less than 0.05), a survival nomogram for predicting prognostic status was constructed based on the MLRS system and the above independent clinicopathologic indicators. A calibration plot was used to estimate the consistency between actual observations and nomogram predictions, and concordance index (C-index) was also applied to test and compared the reliability of the prediction.

### Distribution of MLRSs in different clinicopathological characteristics

The distribution of MLRSs in different clinicopathological features was compared via the Wilcoxon test. Subsequently, patients were divided into different subgroups to compare the difference of OS between the low-MLRS and high-MLRS groups in each subgroup by K-M survival analysis.

### Biological function analysis

Based on the LncSEA database, we pooled the above 8 model mrlncRNA to investigate their potential influence in tumor survival and function with the *p* value less than 0.05 [27]. To further explore the related function of the risk models, the differentially expressed genes (DEGs) between the two MLRS groups were identified according to the standard of |logFC|> 0.585 and false discovery rate (FDR) less than 0.05. Protein‒protein interaction (PPI) network among the DEGs was calculated by the STRING database and subsequently re-visualized via Cytoscape version 3.6.2 software. In addition, the top 10 hub DEGs were selected with the application of cytoHubba. Furthermore, to explore the potential biological functions about these DEGs, gene ontology (GO) and Kyoto Encyclopedia of Genes and Genomes pathway (KEGG) enrichment analyses were conducted via the “clusterProfiler” and “bioconductor” R packages. Furthermore, gene set enrichment analysis (GSEA) was performed to investigate the pathways enriched in the MLRS groups via the GSEA software with the assistance of a related gene set. The eligible pathways in the two MLRS groups were selected while the FDR was less than 0.05.

### Exploration of the relationship of MLRS, tumor mutation burden (TMB), and stemness

The relationship between MLRS and TMB was explored with the application of the “limma” and “matftool” R packages. The Wilcoxon signed-rank test was used to compare the mutation frequencies of the top 20 genes in the low-MLRS and high-MLRS groups, and the survival analysis referring to TMB plus MLRS was also evaluated. In addition, the correlation between MLRS and stem cell-like features, including DNA stem score (DNAss) and RNA stem score (RNAss), was conducted by the use of the Spearman test.

### Assessment of the tumor immune infiltrated microenvironment and clinical treatment

To further assess the TIME, immune-related analyses, including immune cell infiltration, immune function activation, TME scores, and expression of immune checkpoint-related genes, were conducted and compared between the two MLRS groups. Across them, immune cell infiltration status was assessed by multiple algorithms obtained from the TCGA-pancancer dataset. Correlation analysis was conducted based on Spearman’s test, and the results are summarized in the bubble plot. In addition, immune-related analysis (including cells and functions) was also assessed by using the technology of ssGSEA. Furthermore, TME scores, consisting of immune scores, stromal scores, ESTIMATE scores, and tumor purity, were calculated for each sample in the TCGA-HNSC cohort with the “estimate” R package.

The expression of immune checkpoint genes was compared between the low-MLRS and high-MLRS groups to predict the potential immunotherapeutic response. Additionally, the differences in immunotherapy between the two MLRS groups were predicted and compared concerning the immunophenoscore (IPS) from the TCIA database. In addition, the drug sensitivity of HNSCC patients to five commonly used chemotherapeutic agents was evaluated according to the half-maximum inhibitory concentration (IC50) values.

### Identification of tumor subtypes based on the model mrlncRNAs

To further identify the tumor subtypes and assess the TIME, patients of the TCGA-HNSC were then grouped into different clusters with the application of the “ConsensususClusterPlus” R package. Principal component analysis (PCA) and t-distributed stochastic neighbor embedding (t-SNE) analysis were used to assess the distribution about clusters, and survival comparison TIME analysis and immunotherapy prediction were also investigated.

## Results

### Obtaining the survival-related mrlncRNAs and constructing the risk model

**Fig. 1:**
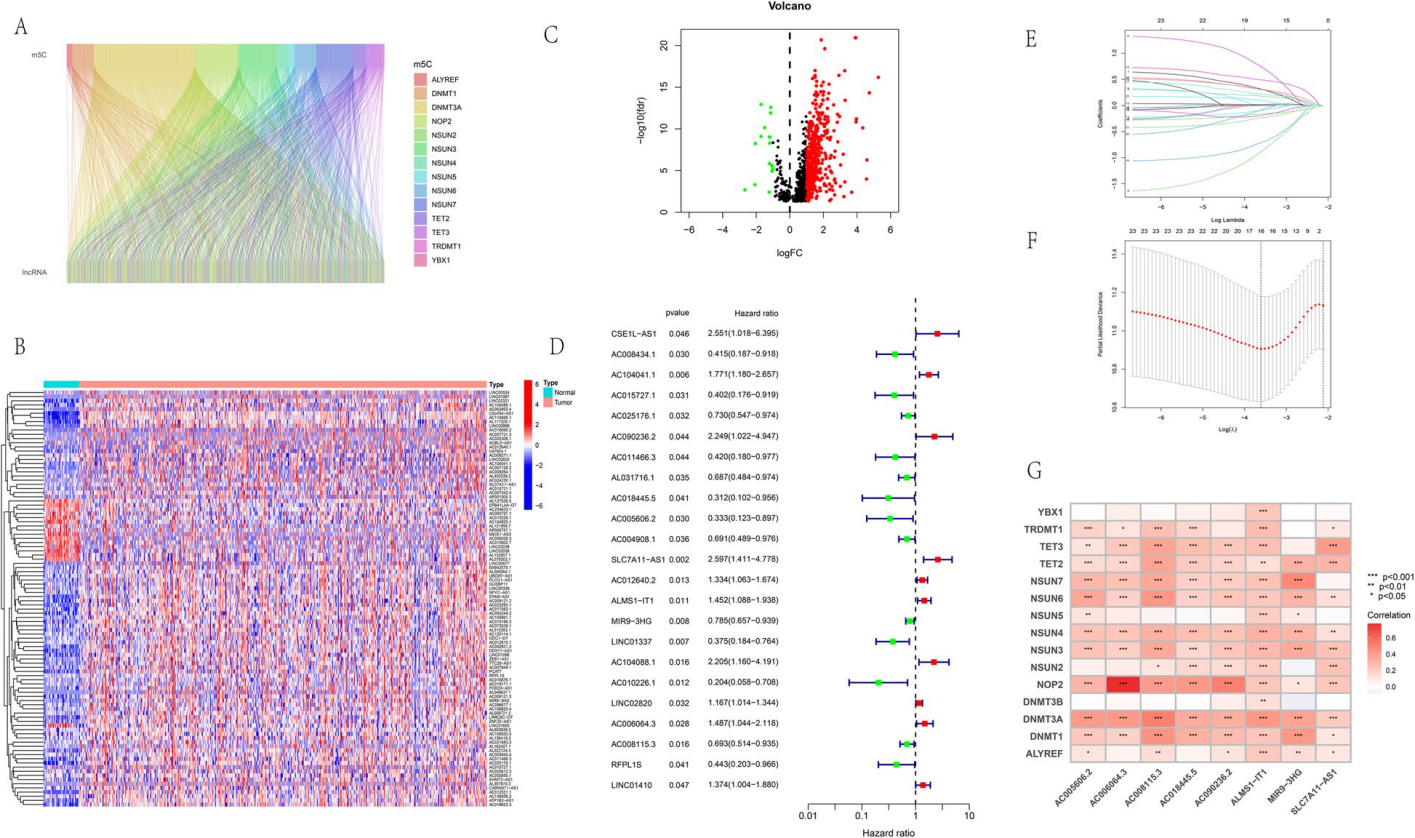
Establishing a prognostic risk model. **A** Sankey diagram to reflect the relationship between m^5^C genes and related lncRNAs. **B** Heatmap about differential expression of mrlncRNAs between tumor and normal samples. **C** Volcano diagram about differential expression of mrlncRNAs. **D** Hazard forest to identify the prognostic mrlncRNAs. **E** LASSO diagram for mrLncRNAs. **F** Cross-validation curve for prognostic mrLncRNAs. **G** Correlation between the model lncRNAs and m^5^C genes

**Fig. 2:**
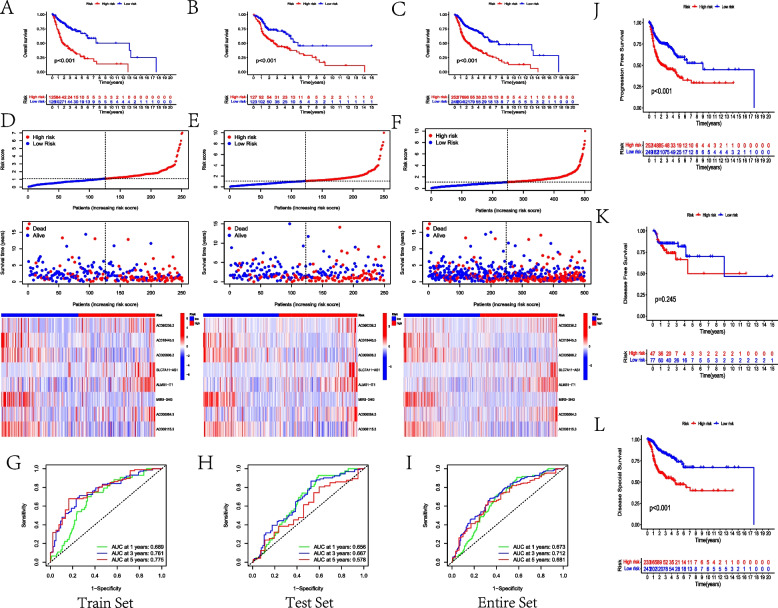
Verification of the risk model. **A**–**C** **K**-**M** survival analysis to compare the OS between the low-MLRS and high-MLRS groups in the train set (**A**), test set (**B**), and entire (**C**) set, respectively. **D**–**F** Exhibition of risk scores, survival time and status, and distributing heatmap of model mrlncRNA in train (**D**), test (**E**), and entire (**F**) sets; **G**–**I** 1-, 3- and 5-year ROCs for the train set (**G**), test set (**H**), and entire (**I**) set, respectively. Comparison of PFS (**J**), DFS (**K**) and DSS (**L**) between the two MLRS groups

Referring to the results of the Pearson correlation test, 865 mrlncRNAs were identified with the criteria of |Pearson R|> 0.04 and *p* value < 0.001 (Fig. 1A–C). Among them, 24 mrlncRNAs were considered survival-related biomarkers in the HNSCC cohort, as shown in the forest plots (Fig. 1D). Subsequently, after selecting the eight model mrlncRNAs according to LASSO regression analysis (Fig. 1E, F), the MLRS risk model was constructed using the following formula: MRLS = AC090236.2 × 1.47873598876185—AC018445.5 × 1.80937632353846—AC005606.2 × 1.0530320771527 + SLC7A11-AS1 × 0.825908137678011 + ALMS1-IT1 × 0.501505889430741—MIR9-3HG × 0.264819137856731 + AC006064.3 × 0.540914230120973—AC008115.3 × 0.396968136166697. The model mrlncRNAs were expressed differently between the normal and HNSCC samples. In addition, the high expression of AC018445.5, AC005606.2, MIR9-3HG, and AC008115.3 displayed better prognosis, whereas the increasing expression of the remaining model mrlncRNAs shortened the OS for patients. The correlation between the model lncRNAs and m^5^C-related genes is shown in Fig. 1G, and the relationship and distribution of MLRS, OS, and signature mrlncRNAs in the train, test, and entire sets are reflected in Fig. 2A–F as well as the K-M survival analysis of OS and ROC curves (Fig. 2G–I). The AUC values for the prognostic ROC curve of the MLRS system in the entire set were 0.673, 0.712, and 0.681, respectively, which were much higher than those of the other clinical features (Fig. 2I). The comparison of PFS, DFS, and DSS between the low-MLRS and high-MLRS groups in the entire cohort was compared, which indicated that patients in the low-MLRS group had longer PFS and DSS but similar DFS to those in the high-MLRS group (Fig. 2J–L).

**Fig. 3:**
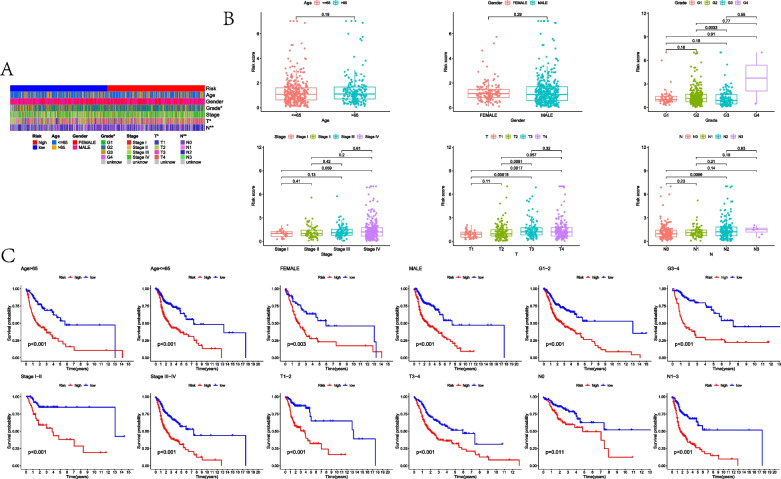
Distribution of MLRS in clinicopathological features and subtype survival analysis. **A** Heatmap about MLRS distributed in clinicopathological features. **B** Boxplots about MLRS distributed in clinicopathological features. **C** Subtype survival analysis in different clinicopathological features

Moreover, when comparing the MLRSs in different clinicopathological features, there were significant differences among different T or N stages (Fig. 3A, B). In addition, as indicated by the subgroup survival analysis, the low-MLRS groups always presented a better prognosis in different clinical subtypes (Fig. 3C).

### Construction and validation of a predictive nomogram

**Fig. 4:**
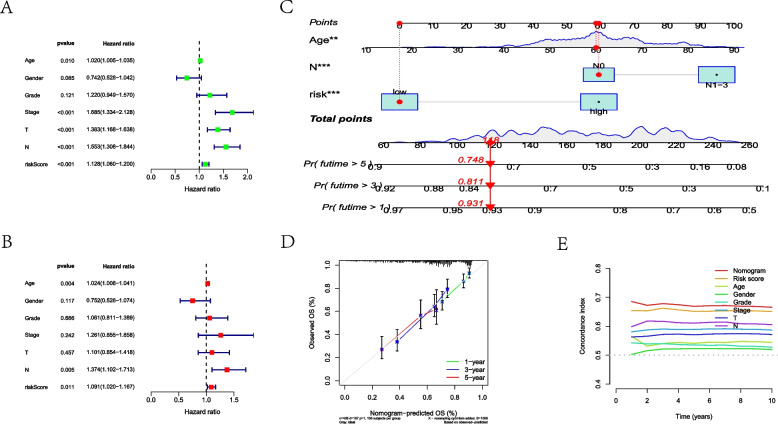
Identifying the independent indicators and establishing a nomogram. **A** Forest plot of the uni-Cox regression analysis. **B** Forest plot of the multi-Cox regression analysis. **C** Nomogram to predict the 1-, 3-, and 5-year prognosis. **D** Calibration curves plot. **E** C-index to evaluate the predictive effects of a nomogram

As indicated by the uni-Cox and multi-Cox regression analyses, the clinicopathological characteristics of A and B were considered independent indicators, as well as MLRSs, with both statistical differences (Fig. 4A, B). Subsequently, the predictive nomogram was established for survival prediction, as shown in Fig. 4C. The calibration and C-index plots suggested high consistency and exhibited satisfactory predictive effects for HNSCC patients (Fig. 4D, E).

### Functional enrichment analysis

Referring to the results of the LncSEA database, eight kinds of tumors (including HNSCC) were identified correlated with these mrlncRNAs in tumor survival. Additionally, in the calculating module of experimentally validated function, these mrlncRNAs were strongly associated with cancer progression and cell migration (Suppl. Fig. 1). In addition, it was also found that SLC7A11-AS1 was involved in three kinds of competing endogenous RNAs, which was related to HNSCC (Suppl. Table 1).

**Fig. 5:**
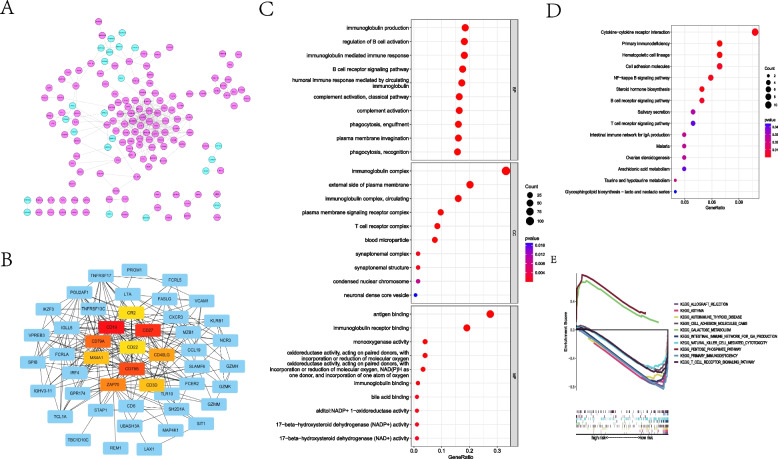
Functional enrichment analysis. **A** Protein–protein interaction network of DEGs between the low- and high-MLRS groups, the purple cycles indicate genes upregulated in the low-MLRS group. **B** Hub gene network of DEGs. **C** GO enrichment analysis. **D** KEGG signaling pathway analysis. **E** GSEA enrichment analysis

According to the selection criteria, 465 DEGs between the two MLRS groups were identified, and the PPI network was re-visualized by Cytoscape software. Blue circles suggested DEGs upregulated in the low-MLRS group, whereas pink circles were more highly expressed in the high-MLRS group (Fig. 5A). A hub gene network was subsequently established, showing the 10 genes with the topmost interactions with other DEGs, including CD19, CD27, CD79B, CD79A, ZAP70, CD40LG, CD3D, MS4A1, CR2, and CD22 (Fig. 5B). The GO analysis indicated that these DEGs were mostly enriched in biological processes, including immunoglobulin production, regulation of B cell activation, B cell receptor signaling pathway, and other immune-related biological progress (Fig. 5C). KEGG determined that these DEGs were mostly enriched in the signaling pathways of cytokine‒cytokine receptor interactions, primary immunodeficiency, and other immune-related signaling pathways (Fig. 5D). Similarly, referring to the criteria of FDR < 0.05, GSEA also determined that the low-MLRS group exhibited enrichment of the immunotherapeutic response; nevertheless, the high-MLRS group was more activated in galactose metabolism and the pentose phosphate pathway (Fig. 5E).

### Exploring the correlation of the MLRS with TMB and stemness

**Fig. 6:**
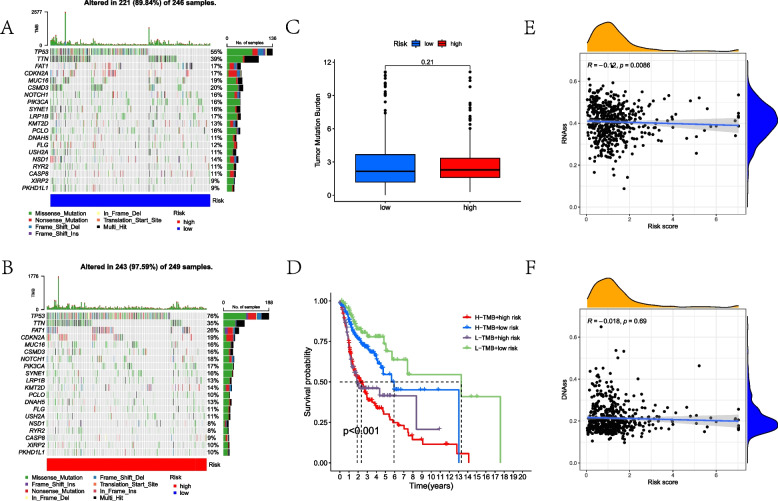
Correlation of MLRS with TMB and stemness. **A** Waterfall plot about the topmost 20 mutated genes in the low-MLRS group. **B** Waterfall plot about the topmost 20 mutated genes in the high-MLRS group. **C** Comparison of TMB between the two MLRS groups. **D** Survival analysis combing with MLRS and TMB. **E** Spearman correlation analysis of MLRSs and stem cell RNA scores. **F** Correlation between MLRSs and DNA scores

The distribution of mutated frequency of the topmost 20 mutated genes from the TCGA-HNSC cohort was reflected in the waterfall plots (Fig. 6A, B), which indicated no significant differences between the low-MLRS and high-MLRS groups (Fig. 6C). Although there was no statistical association between MLRS and TMB, when combining the TMB and MLRS to conduct the survival analysis, those with high TMB and high MRLS displayed the worst prognosis because both increasing TMB and MLRS can enhance the risks for HNSCC patients (Fig. 6D). Moreover, Spearman analysis indicated that MLRS was positively associated with RNAss, whereas there were no statistical differences of correlation test between the MLRS and DNAss (Fig. 6E–F).

### Assessment of the TIME

**Fig. 7:**
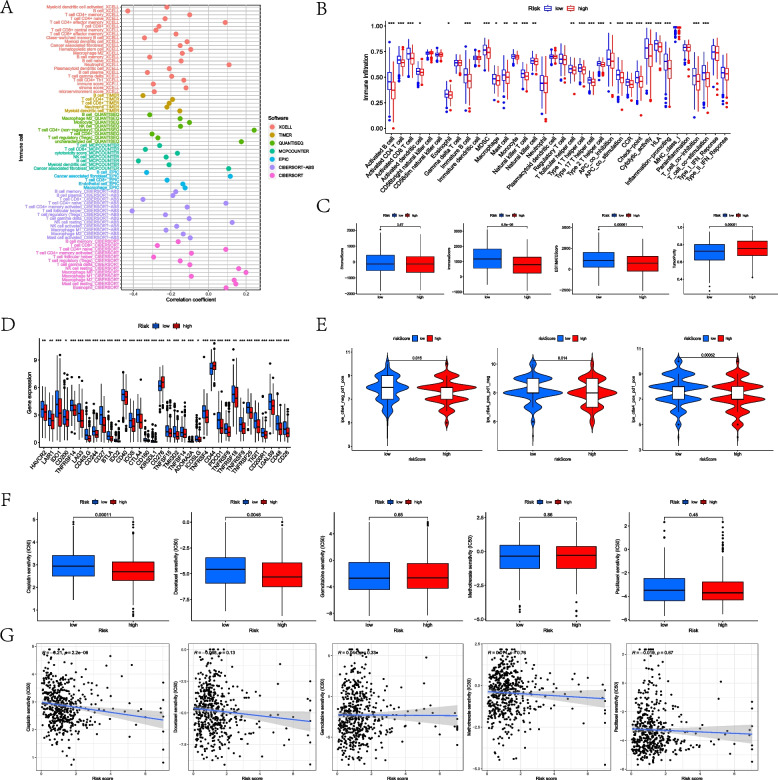
Assessment of tumor immune microenvironment. **A** Immune cell infiltration status based on multiple platforms. **B** Immune cells infiltration and function activation based on ssGSEA methods. **C** TME scores according to estimate platform. **D** Comparative expression of ICI-related genes between the low- and high-MLRS groups. **E** Assessment of immunotherapeutic response about PD-1 and CTLA4 in the two groups. **F** Comparison of IC50 about five chemotherapeutic agents between the MLRS groups. **G** Correlation analysis of IC50 values and MLRSs for five drugs

According to different analysis platforms, the MLRS was negatively associated with most immune cells, including B cells and CD8 + T cells. However, there was a positive association of MLRS with neutrophils and eosinophils infiltration (Fig. 7A). Similar results were also supported by the ssGSEA, in which patients in the low-MLRS group exhibited more activated CD4 + and CD8 + T cell enrichment. Moreover, for those in the low-MLRS group, HNSCC patients exhibited more activation of immune-related functions, such as APC coinhibition, APC costimulation, and CCR (Fig. 7B). Furthermore, for TME scores, patients in the low-MLRS group had higher immune scores and ESTIMATE scores and lower tumor purity than those in the high-MLRS group; nevertheless, for stromal scores, the comparison exhibited no significant differences (Fig. 7C).

### Immunotherapy and chemotherapy

To further assess and predict the immunotherapeutic response of patients to ICI therapy, the expression of ICI-related genes was compared between the low-MLRS and high-MLRS groups. As indicated by Fig. 7D, patients with lower MLRSs showed higher expression of most ICI-related genes, including CTLA4 and PDCD1. The comparison of IPS based on the TCIA database also indicated that the low-MLRS groups performed higher IPS while being treated with PD-1 or CTLA4 inhibitors (Fig. 7E).

Similarly, the potential drug sensitivity to five common chemotherapeutic agents between the two MLRS groups were also assessed and compared. As reflected by the boxplots, patients in the high-MLRS groups had lower IC50 values for cisplatin and docetaxel and were recognized to exhibit higher sensitivity to the chemotherapy of the two drugs. However, the IC50 values of the remaining three drugs behaved similarly, with *p* values > 0.05 (Fig. 7F, G).

### Identifying the hot and cold tumor subtypes for HNSCC

**Fig. 8:**
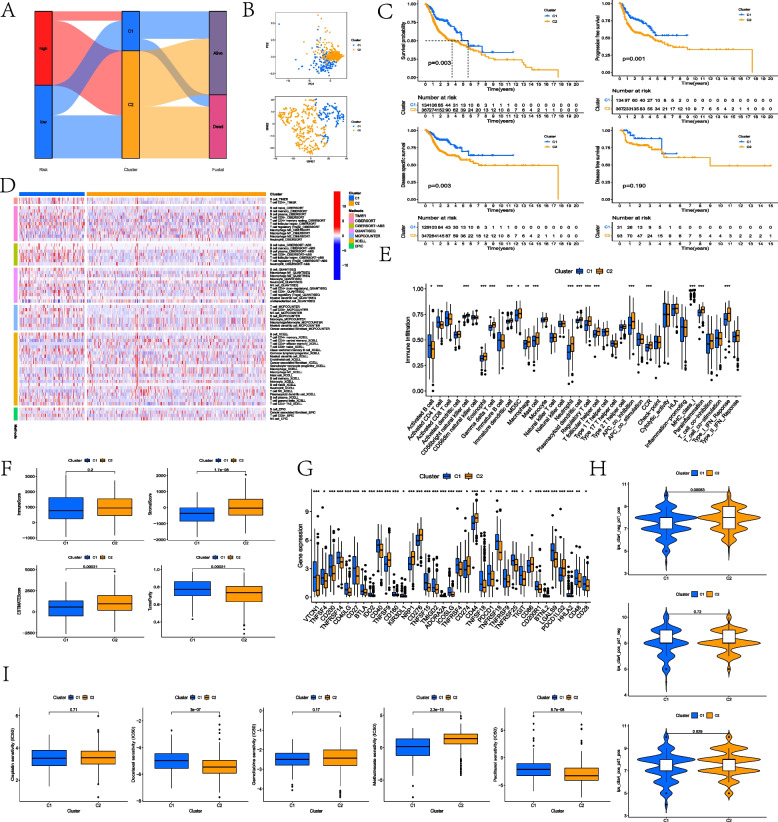
Identifying hot and cold tumor subtypes based on model mrlncRNAs. **A** Sankey diagram to reflect the relationship of MLRS groups, tumor subtypes, and survival status. **B** PCA and t-SNE analysis in clusters. **C** Survival analysis about OS, PFS, DSS, and DFS in clusters. **D** Heatmap about immune cell-infiltrated status in clusters. **E** Immune-infiltrated status based on ssGSEA. **F** TME scores in clusters. **G** Comparison of ICI-related gene expression in clusters. **H** Comparison of IPS in clusters. **I** Assessment of chemotherapy between the two clusters

Referring to the expression of eight model mrlncRNAs, patients were discriminated as two different tumor subtypes, and the Sankey diagram suggested that most of the HNSCC patients in the high-MLRS group were identified and distributed into cluster 2 (Fig. 8A). The PCA and t-SNE analysis indicated that patients of the TCGA-HNSC cohort can be distinguished clearly as two different clusters by these eight model mrlncRNAs (Fig. 8B). As recommended by the survival comparisons, patients in cluster 2 behaved worse OS, PFS, and DSS than the cluster 1; nevertheless, these two clusters exhibited similar DFS according to the comparison (*p* value > 0.05) (Fig. 8C). The immune infiltration-related analysis determined that cluster 1 displayed an immunosuppressive status with less immune cell infiltration, less immune function activation, lower stromal scores, and lower ESTIMATE scores but higher tumor purity (Fig. 8D–F). In addition, cluster 2 appeared much higher expression of ICI-related genes (CD274 and PDCD1LG2), indicating that cluster 2 may be more sensitive to PD-1 inhibitors (Fig. 8G). Similar results were also obtained by comparing IPS; cluster 2 had a higher IPS when treated with either PD-1 or CTLA4 antibodies (Fig. 8H). In addition, cluster 2 behaved higher sensitivity to the drugs of docetaxel and paclitaxel whereas cluster 1 exhibited better chemotherapeutic response in methotrexate (Fig. 8I).

## Discussion

Multiple studies have determined lncRNAs can be regulated by m^5^C-related to participate in the biological process of tumors by regulating of RNA localization, stability, and transcription efficiency [28]. Referring to the summarized list reviewed by Cusenza et al., a large number of lncRNAs have been verified as signatures modified by m^5^C in malignancies, especially for squamous cell neoplasms [29]. For HNSCC patients, a reliable signature for prognostic prediction and immune infiltration assessment is necessary to develop an individual and precise treatment [3]. Based on the results of this study, a novel MLRS system with eight prognostic mrlncRNAs was established in order to conduct a comprehensive evaluation. Among these model mrlncRNAs, lncRNA of ALMS1-IT1 can accelerate tumor malignant progression (e.g., lung adenocarcinoma) via AVL9-mediated activation of the cyclin-dependent kinase pathway [30]. In addition, as indicated by previous evidence-based analysis, it was also identified as one of four lncRNAs for survival prediction of HNSCC [31]. As for SLC7A11-AS1, it can confer malignant progression by repressing miR-4775 and TRAIP expression in lung cancer and reduce tumor growth via the ASK1-p38 MAPK/JNK pathway in gastric cancer [32–34]. Besides, this lncRNA is involved in the cisplatin resistance for gastric tumor with downregulated expression via the SLC7A11-AS1/xCT axis [35]; nevertheless, downregulation of SLC7A11-AS1 can significantly decrease the NRF2/SLC7A11 expression and inhibit the progression of colorectal cancer [36]. And as investigated by Yang et al., these lncRNAs can promote chemoresistance by blocking SCF^b−TRCP^-mediated degradation of NRF2 in pancreatic cancer [37]. While knocking down the MIR9-3HG, in cervical cancer, the proliferation of tumor cells will be inhibited and the apoptosis can be promoted via the EP300 [38]. Similarly, MIR9-3HG can promote carcinogenesis of squamous cell carcinoma by affecting LIMK1 mRNA and protein levels via sponging miR-138-5p and recruiting TAF15, and it was also considered a predictive biomarker in HNSCC via multiple machine learning studies and q-RT PCR [39–42]. Based on previous studies and the results from the LncSEA database, the model mrlncRNAs are considered specifically related to HNSCC and contribute important roles in tumors.

As indicated by our risk model, patients who were assessed with low MLRSs displayed better prognoses in OS, PFS, and DFS, which indicated that increasing MLRS may enhance the risk and shorten the survival time for patients. Similarly, as supported by the results of K-M survival analysis in different clinicopathological subtypes, HNSCC patients with lower MRLSs also showed a better prognosis than these higher MLRS patients. The AUC values for the 1-, 3- and 5-year ROC curves for the MLRS model revealed much more reliable predictive effects than other clinicopathological characteristics and can be used to establish a predictive nomogram with the highest C-index. In addition, although there was no significant statistical correlation between TMB and the MLRS groups, patients could be predicted precisely with different prognostic states when TMB and MLRS were combined. In addition, the increasing MLRS may reduce the RNAss based on the Spearman correlation analysis, suggesting that the high-MLRS group has fewer stem-like cells. Previous studies have noted that stem-like cells are strongly associated with chemotherapy and are considered the main determinant of drug resistance [43, 44]. Therefore, this correlation analysis can explain the results regarding chemotherapeutic sensitivity that the higher MLRS patients exhibited a better chemotherapeutic response to chemotherapy agents.

Furthermore, as indicated by the functional enrichment analysis, the DEGs between the low-MLRS and high-MLRS groups were associated with biological processes and pathways of the immune response. Similarly, GSEA also supported the results that low MLRS was enriched and associated with immune-related biological processes. In addition, the comparison of TIME, including immune cells, functions, and related scores, determined that those in the low-MLRS group revealed more sensitivity to immunotherapy. For patients with low MLRSs, much more CD8 + T cell infiltration promotes better cancer cell killing and immune tolerance disruption [45, 46]. This can be determined by the comparisons of TCIA that the low-MLRS exhibited higher IPSs when treated by the PD-1 inhibitors, and the low-MLRS exhibited higher expression of ICI-related genes (e.g., CD274).

In addition, while regrouping patients into novel tumor subtypes referring to the prognostic model mrlncRNAs, those in cluster 1 had a better prognosis but immunosuppressive status, which resulted in less immune cell infiltration and lower stromal scores. Patients in cluster 2, as determined by the TCIA databases, were more sensitive to immunotherapy and can be considered the hot tumor subtype [47, 48]. Therefore, while being diagnosed with HNSCC, our risk model can assess their risks and identify the tumor type clearly as well as provide detailed immunotherapeutic treatment for those considered hot tumors with a poor prognosis.

However, although our predictive model performed satisfactorily, there were still several limitations in our study. As a predictive model, there is a lack of external lncRNA cohorts to verify the predictive effects. Prospective studies with experimental assays and clinical information are necessary and crucial for further exploration and verification. Actually, we built a model based on m5C-related lncRNAs in TCGA-HNSC cohort and validated it internally based on random allocation, which enhanced the reliability of our results. In addition, we also investigated its predictive value in immune infiltration and immunotherapy based on various algorithms. The coinciding tendency proved our finding also serves as a treatment response indicator and indirectly demonstrated the reliability of this predictive tool. Besides, we applied multiple methods to assess the biological functions, TIME, and clinical therapy to laterally and externally test the prediction, and these results coincided and can be mutually corroborated. Hence, this mrlncRNA risk model can be considered useful and reliable for prognostic prediction.

## Conclusions

Referring to the above results, we established a m^5^C-related lncRNA model to evaluate the prognosis, TME, TMB, and clinical treatments for HNSCC patients. This novel assessment system is able to precisely predict the patients’ prognosis and identify hot and cold tumor subtypes clearly for HNSCC patients, providing ideas for clinical treatment.

## Supplementary Information

**Additional file 1:** **Fig. S1.** Results for tumor survival and experimental validation of model mrlncRNA based on LncSEA database.

**Additional file 2:** **Table S1.** Results for ceRNA in tumor based on LncSEA database.

## Data Availability

Data was openly obtained from The Cancer Genome Atlas and The Cancer Immunome Atlas and available from the corresponding author upon request.

## Abbreviations

HNSCC: Head and neck squamous cell carcinoma
ICI: Immune checkpoint inhibitor
TIME: Tumor immune microenvironment
m^5^C: 5-Methylcytosine
lncRNA: Long non-coding RNA
mrlncRNA: M^5^C-related lncRNA
TCGA: The Cancer Genome Atlas
OS: Overall survival
uni-Cox: Univariate Cox
multi-Cox: Multivariate Cox
LASSO: Least absolute shrinkage and selection operator
MLRS: M^5^C-related lncRNA risk score
K-M: Kaplan‒Meier
PFS: Progression-free survival
DFS: Disease-free survival
DSS: Disease-specific survival
AUC: Areas under the curve
ROC: Receiver operating characteristic
C-index: Concordance index
DEG: Differentially expressed gene
FDR: False discovery rate
GO: Gene ontology
KEGG: Kyoto Encyclopedia of Genes and Genomes pathway
GSEA: Gene set enrichment analysis
TMB: Tumor mutation burden
DNAss: DNA stem score
RNAss: RNA stem score
IPS: Immunophenoscore
IC50: Half-maximum inhibitory concentration
PCA: Principal component analysis
t-SNE: T-Distributed stochastic neighbor embedding
