# Supplementary figures and images for: 5-Methylcytosine-related lncRNAs: predicting prognosis and identifying hot and cold tumor subtypes in head and neck squamous cell carcinoma
Source: World J Surg Oncol. 2023 Jun 14;21:180. doi: 10.1186/s12957-023-03067-w (PMC10265901; doi:10.1186/s12957-023-03067-w)

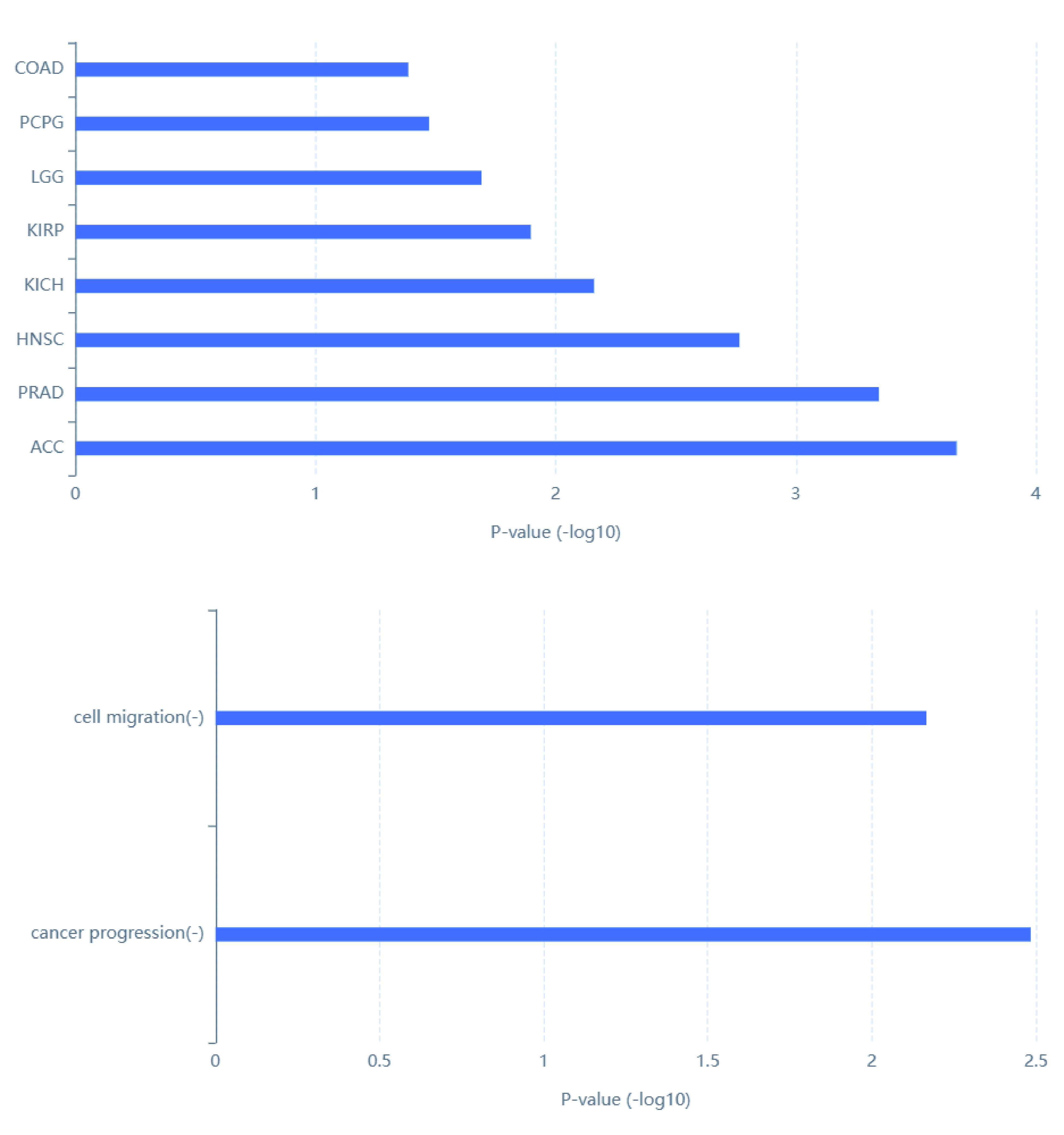

Supplement: Supplementary file 1 — Additional file 1: Fig. S1. Results for tumor survival and experimental validation of model mrlncRNA based on LncSEA database. [file 12957_2023_3067_MOESM1_ESM.jpg]
